# Adult-onset Still’s disease complicated by macrophage activation syndrome during pregnancy: a case-based review

**DOI:** 10.1007/s10067-023-06664-y

**Published:** 2023-06-15

**Authors:** Leanna Wise, Monica Zell

**Affiliations:** 1 Division of Rheumatology, Department of Internal Medicine, Keck School of Medicine, University of Southern California, 2020 Zonal Ave. IRD 431, CA 90033 Los Angeles, USA; 2 Department of Internal Medicine, Keck School of Medicine, University of Southern California, Los Angeles, USA

**Keywords:** Critical illness, Cytokines, Immunosuppression therapy, Macrophage activation syndrome, Pregnancy, Still’s disease, adult-onset

## Abstract

Adult-onset Still’s disease is a rare, systemic inflammatory rheumatic disease characterized by recurrent fevers, arthritis, and an evanescent rash. One of the most serious hematologic derangements that can be seen with adult-onset Still’s disease is macrophage activation syndrome. Macrophage activation syndrome is characterized by activation of lymphocytes, resulting in a cytokine storm and hemophagocytosis in the bone marrow, along with multi-organ failure. Adult-onset Still’s disease with macrophage activation syndrome first presenting during pregnancy is exceptionally rare; here, we report two unique cases of such a presentation and review the pertinent literature. Both of our cases presented critically ill with end-organ failure, and responded to immunosuppression; fetal demise was present in one and an emergency caesarean section with a viable fetus was performed in the other patient. Maternal outcomes were favorable in both cases and both patients did well long-term with systemic therapy. Systemic immunosuppression, particularly anti-IL1 therapy, may be considered as treatment for this rare and life-threatening condition when presenting during pregnancy.

## Introduction

Adult-onset Still’s disease (AoSD) is a rare autoinflammatory syndrome that can present with macrophage activation syndrome (MAS), a hyperinflammatory state resulting in pancytopenia, coagulopathy, and multi-organ failure. While AoSD largely tends to affect individuals under the age of 50, reports of AoSD with MAS during pregnancy are few; both timely diagnosis and treatment can be challenging in this population. To our knowledge, only two cases of AoSD with MAS first presenting during pregnancy have been reported in the literature. In this case-based review, we present two patients with newly diagnosed AoSD/MAS during pregnancy, and we describe their clinical courses and outcomes. We then review the pertinent literature on MAS and pregnancy, with a focus on the utilization of anti-IL1 therapies.

## Methods

The local institutional review board approved this study (HS-23-00135). This is a case-based review of two patients, with a review of previously reported cases in the literature. Both patients described in this study gave consent for their cases to be published. In describing each case report, we adhered to consensus guidelines for clinical case reports (CAse REport; CARE guidelines) [1]. For determination of prior case reports, we performed a review of Medline/PubMed from January 1977 to February 2023 with no restriction on language. We used the following words in various combinations and all synonyms of the MeSH terms “pregnancy,” AND “connective tissue disease,” OR “systemic lupus erythematosus,” OR “adult-onset Still’s Disease,” AND “macrophage activation syndrome,” OR “hemophagocytic lymphohistiocytosis.” We also searched for other cases by reviewing the references of relevant papers. Case reports that explicitly noted pregnancy in the context of a systemic rheumatic disease with concurrent macrophage activation syndrome and/or hemophagocytic lymphohistiocytosis were included.

## Case no. 1

A 21-year-old nulliparous woman with no medical history presented at 22 weeks gestation to a regional hospital with complaints of several weeks of fever, arthritis, diffuse abdominal pain, weakness, and fatigue. Upon admission, the patient was tachycardic and had daily low-grade fevers until hospital day (HD) 3, when they increased in magnitude and persisted despite broad-spectrum antimicrobial coverage. Imaging ruled out pulmonary embolism. On HD4, preterm premature rupture of membranes resulted in the spontaneous vaginal delivery of a non-viable fetus of 22 weeks and 4 days of gestational age. Placental pathology from the outside hospital disclosed moderate villus edema and focal mild acute chorioamnionitis. Retained products of conceptions were ruled out. Laboratory results showed worsening anemia, thrombocytopenia, cholestatic hyperbilirubinemia, and coagulopathy. Given the concern for liver failure, she was transferred to our liver transplant center for higher level of care. On the day of transfer, methylprednisolone 80-mg IV dosed every 8 hours was ordered; the patient presumably received two doses prior to transfer.

On admission to our hepatology service, she was afebrile, and had mild scleral icterus, decreased breath sounds at the bases bilaterally, diffuse abdominal pain, and anasarca on exam. Biochemical analysis on arrival to our institution demonstrated marked transaminitis, pancytopenia, and high ferritin (Table 1). After arrival to our institution the patient became persistently febrile to a maximum of 103°F and rapidly decompensated with acute respiratory failure and lactic acidosis. She was transferred to the medical intensive care unit and intubated, and started on vasopressor support along with continuous renal replacement therapy (CRRT) given her hypotension and metabolic acidosis. Pertinent negative infectious studies included blood and urine cultures, and extensive viral and bacterial workup. Antinuclear antibody (ANA) and rheumatoid factor (RF) were negative. Computed tomography (CT) of the chest, abdomen, and pelvis showed pericardial effusion, pulmonary edema, bilateral pleural effusions, diffuse anasarca, ascites, hepatosplenomegaly, and lymphadenopathy.

**Table 1:** Biochemical findings for case no. 1 and case no. 2

Significant nadir or peak biochemical analyses at time of admission to our institution (units; normal range)	Case no. 1	Case no. 2
WBC (mm3; 4.1–10.9)	0.25	5.49
Hgb (g/dL; 11.7–15.7)	7	8.6
Hct (%; 34.9–46.9)	20.4	26.6
Plt (mm3; 150–400)	25	210
Ferritin (ng/mL; 5–204)	82,627	>40,000
AST (units/L; 0–32)	3570	637
ALT (units/L; 0–33)	454	192
INR (0.9–1.1)	4.1	1.3
CRP (mg/L; ≤4.9)	23.8	15
ESR (mm/h; 0–20)	NR	8
LDH (units/L; 135–214)	7,370	1,831
Fibrinogen (mg/dL; 195–460)	136	124
TGs (mg/dL; ≤149)	225	465
Aldolase (units/L; 1.5–8.1)	NR	29
Functional NK cells (LU30; 7–125)	0	2
Soluble IL-2 receptor/CD25 (pg/mL; <1033)	23,360	13,582

*WBC*, white blood cell count; *Hgb*, hemoglobin; *Hct*, hematocrit; *Plt*, platelet; *AST*, aspartate transaminase; *ALT*, alanine transaminase; *INR*, international normalized ratio; *CRP*, C-reactive protein; *ESR*, erythrocyte sedimentation rate; *LDH*, lactate dehydrogenase; *TGs*, triglycerides; *NK*, natural killer; *IL*, interleukin; *NR*, not reported

In light of her rapid deterioration, fever of unknown origin, multi-organ failure, and intravascular coagulopathy, the possibility of hemophagocytic lymphohistiocytosis (HLH) vs. MAS secondary to an underlying systemic rheumatic disease was considered. While the patient was intubated, her family noted that the patient had complained of ongoing fatigue, poor weight gain during pregnancy, frequent joint pain, and a transient rash on the trunk and extremities. After obtaining this further history, AoSD with possible concurrent MAS was high on the list of differential diagnoses.

Our rheumatology service initiated a course of methylprednisolone 500-mg IV daily for the empiric treatment of a systemic inflammatory syndrome. Within 12 h of receiving her first dose, her vital signs began to rapidly improve. She underwent a bone marrow biopsy, which revealed a hypercellular marrow with markedly increased histiocytes with hemophagocytosis. At this time, a diagnosis of MAS was made based on clinical and biochemical evidence. Further studies later revealed a markedly elevated soluble interleukin-2 receptor (CD25) level of 23,360 pg/mL (normal <1033 pg/mL) and a low absolute natural killer (NK) cell count of 4 (normal range 78–470), signifying that the patient met all eight criteria for HLH, although NK cell enumeration was measured rather than activity [2]. A later mutational analysis was negative for 21 tested genes associated with hereditary HLH.

After receiving 2 days of methylprednisolone, she was initiated on an 8-week induction HLH-94 protocol (etoposide 150mg/m^2^, which was 50% dose-reduced due to hyperbilirubinemia, twice per week for 2 weeks followed by once per week for 6 weeks, plus dexamethasone 20-mg IV daily for 2 weeks followed by a taper). The patient’s laboratory derangements dramatically improved after the first days of treatment, with eventual resolution of lactic acidosis, and improvements in transaminases, ferritin, LDH, and coagulation studies. She was weaned off vasopressors, successfully extubated after 5 days on ventilatory support, and had full renal recovery.

The patient met three of four major Yamaguchi criteria for AoSD (typical salmon-colored rash, seen by the authors on photos from the patient’s cellphone, arthralgias/arthritis, and leukocytosis; subjective fevers had only started just prior to presentation), and five of five minor Yamaguchi criteria (sore throat, lymphadenopathy, hepatomegaly/splenomegaly, elevation in liver enzymes, and RF and ANA negative), although several of these can independently occur in HLH/MAS without AoSD [3]. No other etiology other than AoSD was found to explain the persistence and full constellation of her symptoms.

After the completion of 8 weeks of induction therapy, there was evidence of persistent hepatic injury and rising ferritin levels. Oral cyclosporine was started and titrated to trough level of 200–400. She continued to be followed closely at our institution and completed the 24 weeks of HLH-94 protocol with appropriate response. Our hematology service deemed her a transplant candidate, and she was thus admitted for bone marrow harvest stem cell transplantation (SCT). The patient made a full recovery from SCT and subsequently became pregnant with twins. She had an uncomplicated delivery of two healthy baby boys and had a successful singleton pregnancy 2 years later. At time of writing (over 3 years after initial presentation), she has not had any further AoSD or MAS flares and her three children are healthy.

## Case no. 2

A 41-year-old woman with a history of hypertension and non-alcoholic fatty liver disease was admitted to our institution for a higher level of care [4]. She presented to an outside hospital 5 weeks prior with 1 week of daily fevers, chills, nausea and vomiting, and a cough. At that time, she was about 30 weeks pregnant with a baby conceived via in vitro fertilization (IVF), and her pregnancy course had been complicated by gestational diabetes and cholestasis of pregnancy. At time of admission to the outside hospital, she had an extensive infectious and autoimmune workup for her fevers that had been largely negative, although she was noted to have a mild transaminitis and leukocytosis. Due to persistent fevers and rising liver enzymes, she underwent emergency C-section at 33 weeks, and delivered a viable baby boy. She was discharged home but re-presented back to the outside hospital with persistent fevers and worsening transaminitis. A possible obstructing gallbladder stone was seen on imaging, and the patient underwent a laparoscopic cholecystectomy. During this surgery it was noted that her liver appeared “mottled and purple”; an intraoperative wedge liver biopsy was performed. At this time, there was some concern for HLH/MAS and the patient was given one dose of dexamethasone 20-mg IV and transferred to our institution for management of worsening liver failure. Just prior to her transfer, she had a bone marrow biopsy; results from neither this nor her liver biopsy were available at time of transfer.

On arrival to our institution, the patient was hemodynamically stable, but persistently febrile and lethargic on exam. She was oriented to person, place, and time, but was slow to respond to questions and appeared sleepy. Exam was notable for a well-healing C-section scar with no evidence of surrounding infection and no uterine tenderness or vaginal bleeding. She had no retained products of conception. Biochemical analyses at time of admission are noted in Table 1. An extensive viral and bacterial workup was negative, as were blood and urine cultures. Rheumatologic workup including ANA and RF was negative. Genetic testing for familial HLH sequencing or variants was negative. A CT of the abdomen and pelvis revealed an enlarged and heterogeneously enhancing liver, trace ascites, expected post-partum changes of the uterus without evidence of infection, and trace bilateral pleural effusions.

On review of her liver and bone marrow biopsies prior to transfer, hemophagocytosis was seen on liver biopsy, but not on bone marrow biopsy. While the patient initially denied a history of rashes, she noted some arthralgias during the course of her illness, which she attributed to pregnancy. On further discussion with family members after patient consent, the patient’s sister and husband noted an intermittent well-demarcated salmon-colored rash on patient’s neck and chest that appeared during febrile episodes. This rash also occurred while patient was admitted at our hospital.

Given the above constellation of symptoms, physical exam findings, biochemical and imaging evidence, and pathologic reports, a diagnosis of AoSD with MAS was made. The patient met Yamaguchi criteria with at least three of four major criteria (fever for at least 1 week; typical salmon-colored rash; leukocytosis; possible arthritis although unclear in the setting of pregnancy) and three of five minor (hepatosplenomegaly, abnormal liver function tests, negative RF/ANA) criteria. The patient was started on dexamethasone 40-mg IV with rapid clinical and biochemical improvement. She was discharged from the hospital on an oral corticosteroid taper and anakinra 100 mg intramuscular every day. She was eventually weaned off corticosteroids and her anakinra was changed to canakinumab injections monthly for ease of administration. One year after discharge, she remains on canakinumab injections and has had no further evidence of active AoSD or MAS. Her son has been healthy since birth as well, and she has not attempted to conceive again.

## Discussion

Our two cases demonstrate the diagnostic challenges and therapeutic gravity of new-onset AoSD with MAS presenting during pregnancy.

Adult-onset Still’s disease is a rare autoinflammatory rheumatic disease that is characterized by a wide range of systemic symptoms including but not limited to quotidian fevers, an evanescent rash, and arthritis. Concurrent symptoms may include pharyngitis (often preceding other symptoms), lymphadenopathy, serositis, and hepatomegaly [5]. Its estimated incidence is between 0.16 and 0.4/100,000 people, and generally affects women and men equally [6]. It has a bimodal distribution, affecting those between the ages of 15 and 25 as well as those between 36 and 46 years old. AoSD’s non-specific symptoms mandate that it is often a diagnosis of exclusion; the provider must rule out other rheumatologic processes, infection, malignancy, or drug reaction prior to making the diagnosis of AoSD. Quotidian fevers are a classic manifestation of the disease, as are “salmon-colored” rashes that tend to occur at the time of the fever. However, it should be noted that a variety of cutaneous manifestations have been associated with AoSD, including urticaria, diffuse pruritus, plaques, and pustules [7]. Up to 74% of patients may note pharyngitis prior to the systemic illness [6, 8]. Serious and life-threatening manifestations of AoSD include hepatic dysfunction and pulmonary hypertension, as well as macrophage activation syndrome. Laboratory findings in AoSD reflect the underlying systemic inflammatory response, and an elevated ESR, CRP, and extremely high ferritin are common. A variety of diagnostic criteria have been proposed for AoSD, with the Yamaguchi criteria most commonly used (Table 2) once other causes of systemic inflammation have been excluded [3].

**Table 2:** Yamaguchi criteria

Five or more criteria required for diagnosis, with at least two being major criteria				
Major criteria	Fever >39°C for at least 1 week	Arthritis or arthralgia for at least 2 weeks	Leukocytosis greater than 10,000 cells/mm^2^ with at least 80% polymorphonuclear cells	Typical rash (salmon-colored maculopapular non-pruritic rash with febrile episodes)
Minor criteria	Sore throat	Lymphadenopathy and/or splenomegaly	Abnormal liver function tests	Negative tests for RF, ANA
Exclusion criteria	Absence of infections	Absence of malignancies	Absence of other inflammatory/autoimmune conditions	

One the most dreaded hematologic complications associated with AoSD is secondary hemophagocytic lymphohistiocytosis (sHLH), also known as macrophage activation syndrome (MAS), which may occur in up to 10–15% of those with AoSD [9]. MAS is a highly inflammatory condition characterized by an uncontrolled and dysregulated immune response (particularly of T cells and macrophages), culminating in hemophagocytosis, coagulopathy, and a cytokine storm [10, 11]. It has been associated with other rheumatologic diseases such as systemic juvenile idiopathic arthritis and systemic lupus erythematosus (SLE); mortality of MAS in rheumatologic diseases may reach 30–40% [10, 12–14]. MAS can also occur with viral, bacteria, fungal, and parasitic infections, and it has also been observed with hematopoietic malignancies as well as with some solid tumors [15].

Classic findings in MAS are a non-remitting fever (as opposed to the quotidian fever seen in AoSD without MAS), pancytopenia, a paradoxical drop or normalization of the ESR due to hypofibrinogenemia, liver enzyme elevation, hyperferritinemia, and hemophagocytosis on pathologic evaluation of the bone marrow, liver, or lymph nodes [11]. The pathophysiology of MAS is centered on excessive activation and expansion of T lymphocytes and tissue macrophages, resulting in amplified production of cytokines that lead to an inflammatory milieu. Key cytokines in the pathophysiology of both MAS/sHLH and primary HLH include IL-1β, IL-6, IL-18, and interferon-γ [11]. This hyperinflammatory state of MAS can lead to septic shock and multiorgan failure. Options for management of MAS in rheumatologic diseases include glucocorticoids, intravenous immunoglobulin, IL-1 and IL-6 directed therapies, and calcineurin inhibitors [16, 17]. While etoposide and stem cell transplant may be used in primary HLH treatment, they are not routinely used in MAS [18]. As the underlying immune system derangements and pathophysiology of MAS are similar to that of HLH, the criteria for HLH diagnosis (HLH-2004 criteria) are often extrapolated to diagnosis of MAS in rheumatic disease [10, 19]. However, it should be noted that these criteria have not been validated for MAS diagnosis in rheumatic diseases. A scoring system for sHLH/MAS, the HScore, has been explored as a promising alternative option [20]. Similar to HLH diagnostic criteria, the HScore also evaluates clinical (degree of fever and presence of organomegaly), biochemical (presence of cytopenias, degree of ferritin elevation, and degree of fibrinogen and triglyceride depression), and pathologic findings (hemophagocytosis features) to determine the probability of MAS.

Given that AoSD is a rare disease to begin with, data on the outcomes of pregnancy in AoSD have accumulated extremely slowly over the past few decades. A recent review summarized 49 cases in the literature connecting pregnancy and AoSD between 1971 and 2019 [21]. Twenty-two of these individuals experienced first presentation of AoSD during the pregnancy. In this sub-cohort of 22 patients, 76.5% had a poor obstetrical outcome. Only one of these 22 patients had newly diagnosed AoSD with concomitant MAS during the pregnancy; to our knowledge, there have only been two total cases (in addition to ours) of new AoSD with MAS first presenting during the peripartum period in the medical literature [22, 23]. The first case involved a 41-year-old woman with a history of AoSD who was pregnant with twins and diagnosed with MAS at 19 weeks gestational age. She was treated with pulse glucocorticoids and a steroid taper and delivered her twins at 30 weeks due to intrauterine growth retardation. The patient and her twin infants did well post-partum. The second case of AoSD/MAS during pregnancy occurred in a 26-year-old female who was 35 weeks pregnant when she presented with malaise, fevers, myalgias, and abdominal pain, and was found to be febrile, hypotensive, and tachycardic; she had no diagnosis of AoSD prior [23]. Her baby was delivered via C-section. In less than 3 weeks of delivery, the patient developed a new rash, pancytopenia, worsening transaminases, pleural effusions, and inflammatory arthritis; an extensive infectious workup was negative and a presumptive diagnosis of new-onset peripartum AoSD was made. She was treated with oral prednisone and eventually transitioned to anti-IL-6 therapy with tocilizumab. Presentations of MAS due to an underlying rheumatic disease during pregnancy have been documented in a total of 17 cases, including our two cases (Table 3) [22–31]. Maternal demise was observed in one of these cases and fetal demise (outside of therapeutic abortion) occurred in four of these cases.

**Table 3:** Documented cases of pregnancy-related HLH/MAS in the setting of a rheumatologic disease

Author (reference number)	Maternal diagnosis	Maternal age	Gestational age at presentation (weeks)	Treatment	Maternal outcome	Fetal outcome
Perard [22]	SLE	28	22	GCs, IVIg	Alive	Alive
Yoshida [23]	SLE	33	3 weeks post-partum	GCs, TAC	Alive	Alive
Dunn [20]	AoSD	41	19	GCs	Alive	Alive
Hannebicque-Montaine [24]	SLE	29	21	GCs, IVIg	Alive	Alive
Kim [25]	SLE	29	12	GCs, CSA, IVIg, splenectomy	Alive	TAB
Komaru [26]	pSS	36	5.5 weeks post-partum	GCs	Alive	Alive
Rousselin [27]	Likely SLE vs. MCTD	44	30	GCs	Alive	Alive
Parrott [28]	SLE	28	18	GCs, AZA, IVIg, ETOP, CSA	Maternal demise	Fetal demise
Peters [21]	AoSD	26	35	GCs, MTX, TNFi, TCZ	Alive	Alive
Liu Case #2 [29]	AoSD	24	13	CS, IVIg,	Alive	Fetal demise
Liu Case #3 [29]	SLE	23	12	CS, IVIg,	Alive	TAB
Liu Case #4 [29]	SLE	22	24	CS, IVIg,	Alive	Fetal demise
Liu Case #9 [29]	SLE	31	14	CS, IVIg, CSA	Alive	TAB
Liu Case #10 [29]	SLE	27	36	CS, IVIg, ETOP	Alive	Alive
Ren [30]	SLE	22	24	GCs, CSA	Alive	TAB
Case 1	AoSD	21	22	GCs, ETOP, CSA, SCT	Alive	Fetal demise
Case 2 [5]	AoSD	41	28	GCs, anakinra, CAN	Alive	Alive

*SLE*, systemic lupus erythematosus; *NR*, not reported; *GC*, glucocorticoids; *IVIg*, intravenous immunoglobulin; *TAB*, therapeutic abortion; *TAC*, tacrolimus; *AoSD*, adult-onset Still’s disease; *pSS*, primary Sjogren’s syndrome; *MCTD*, mixed connective tissue disease; *AZA*, azathioprine; *ETOP*, etoposide; *CSA*, cyclosporine A; *SCT*, stem cell transplantation; *CAN*, canukinumab

Both the innate and adaptive immune system changes during pregnancy are incredibly complex and a testament to the remarkable phenomenon of pregnancy—that of a semi-allogeneic fetus in a maternal-fetal interface, while the mother (usually) continues to maintain immune homeostasis [32, 33]. Of note, in-depth mechanistic understanding of immune system changes (in both normal and pathologic pregnancies) in the second and third trimesters is difficult due to ethical reasons, including but not limited to the need for excessive phlebotomy in a pregnant patient. Unfortunately, there are no data that delineate whether there is a unique cytokine profile associated with MAS development in a pregnant patient with rheumatic disease compared to its development in a non-pregnant patient. It is interesting that nearly all cases of MAS in pregnant patients occurred in patients with either AoSD (a disease primarily rooted in aberrant innate immune system function) or SLE (a disease primarily of adaptive immunity dysfunction). However, there is no data on potential differences in MAS pathogenesis in a patient with AoSD vs. in a patient with SLE.

Regardless, the gravity of AoSD in pregnancy with associated MAS, along with the need for urgent cytotoxic and immunosuppressive therapy, requires collaborative discussion among consulting obstetricians, hematologists, and rheumatologists to achieve the best fetal and maternal outcomes. Acceptable treatments for MAS in pregnancy are limited due to a variety of factors including the legitimate concern of exposing the developing fetus (or breastfeeding infant, for those patients with active disease after delivery) and the sheer rarity of the condition, resulting in limited data highlighting successful therapies. Corticosteroids, while they may contribute to intrauterine growth restriction, are likely the safest and most fast-acting first-line option.

Given the prominence of IL-1 in the development and propagation of MAS, anti-IL-1 therapies are an attractive option. A recent review evaluated 88 unique pregnancies that occurred with anti-IL-1 exposure (anakinra or canakinumab) in mothers with a range of autoinflammatory disorders [34]. A single fetal demise was noted, along with 2 cases of renal agenesis; nearly 65% of the anti-IL-1 exposed pregnancies resulted in a healthy term delivery with no maternal or fetal complications. Of note, many of these babies were successfully breastfed while continuing to be exposed to maternal anti-IL-1 therapy; no infections were noted. Rilonacept, an anti-IL1 receptor antagonist, is considered pregnancy category C, and to our knowledge no case reports or case series exist regarding its use during pregnancy. While neither of our patients received anti-IL-1 therapy during pregnancy, this may be a reasonably safe option for patients needing more than glucocorticoids to control AoSD/MAS during pregnancy, or in cases where glucocorticoids must be minimized, such as in gestational diabetes. Finally, biologics such as IL-6 inhibitors and tumor necrosis factor (TNF) inhibitors have been used in recalcitrant AoSD outside of pregnancy. Given the increasing body of evidence behind the safety of TNF inhibitors in systemic autoimmune diseases, their use in pregnant patients with AoSD may be warranted, although data supporting their use as an acute or first-line treatment for MAS are lacking, and some case reports note precipitation of MAS by TNF inhibitor use [35, 36]. Intravenous immunoglobulin (IVIg) has also been used with success in the treatment of HLH/MAS during pregnancy, including in presentations outside an underlying rheumatic disease [37].

It should be noted that there are potential mimics of AoSD and MAS in pregnancy. Viral hepatitis may occur during pregnancy and cause systemic symptoms, as well as elevation of liver enzymes. Pre-eclampsia may manifest as hypertension, thrombocytopenia, and both kidney and liver dysfunction. Hemolysis, elevated liver enzymes, and low platelets (HELLP) syndrome is an obstetric emergency that may mimic both AoSD and MAS. Further, acute fatty liver of pregnancy (AFLP) may also mimic MAS as it can present with constitutional symptoms and liver and/or renal failure. The treatment for pre-eclampsia, HELLP, and AFLP is delivery of the baby.

In summary, AoSD in pregnancy may be associated with poor maternal and fetal outcomes. These poor outcomes appear to be magnified by the co-presentation of MAS in addition to active AoSD. Both AoSD and MAS activity during pregnancy pose a challenge given limited data on treatment safety and efficacy. Conversely, good pregnancy outcomes in AoSD are likely underreported in the literature, and well-controlled disease before, during, and after pregnancy (along with uneventful maternal and fetal health outcomes) may not be as uncommon as case reports and case series imply. Ultimately, AoSD with concurrent MAS is a life-threatening occurrence in non-pregnant patients, and when it occurs in pregnancy it may lead to poor maternal and fetal outcomes, such as our first patient’s case. Timely treatment is paramount to optimize the best possible outcomes for both the patient and the fetus, and IL-1–directed therapy may be a very reasonable option to pursue in patients presenting with AoSD (with or without MAS) during pregnancy. We hope that our two descriptions of MAS presenting in pregnant patients with new-onset AoSD, along with our summary of similar cases in AoSD and SLE, will help clinicians understand and manage this rare entity.

## Declarations

### Disclaimer

All the authors take full responsibility for the accuracy and integrity of the data in this manuscript.
